# *Spiroplasma* Bacteria Enhance Survival of *Drosophila hydei* Attacked by the Parasitic Wasp *Leptopilina heterotoma*

**DOI:** 10.1371/journal.pone.0012149

**Published:** 2010-08-13

**Authors:** Jialei Xie, Igor Vilchez, Mariana Mateos

**Affiliations:** Department of Wildlife and Fisheries Sciences, Texas A&M University, College Station, Texas, United States of America; Royal Holloway University of London, United Kingdom

## Abstract

**Background:**

Maternally-transmitted associations between endosymbiotic bacteria and insects are ubiquitous. While many of these associations are obligate and mutually beneficial, many are facultative, and the mechanism(s) by which these microbes persist in their host lineages remain elusive. Inherited microbes with imperfect transmission are expected to be lost from their host lineages if no other mechanisms increase their persistence (i.e., host reproductive manipulation and/or fitness benefits to host). Indeed numerous facultative heritable endosymbionts are reproductive manipulators. Nevertheless, many do not manipulate reproduction, so they are expected to confer fitness benefits to their hosts, as has been shown in several studies that report defense against natural enemies, tolerance to environmental stress, and increased fecundity.

**Methodology/Principal Findings:**

We examined whether larval to adult survival of *Drosophila hydei* against attack by a common parasitoid wasp (*Leptopilina heterotoma*), differed between uninfected flies and flies that were artificially infected with *Spiroplasma*, a heritable endosymbiont of *Drosophila hydei* that does not appear to manipulate host reproduction. Survival was significantly greater for *Spiroplasma*-infected flies, and the effect of *Spiroplasma* infection was most evident during the host's pupal stage. We examined whether or not increased survival of *Spiroplasma*-infected flies was due to reduced oviposition by the wasp (i.e., pre-oviposition mechanism). The number of wasp eggs per fly larva did not differ significantly between *Spiroplasma*-free and *Spiroplasma*-infected fly larvae, suggesting that differential fly survival is due to a post-oviposition mechanism.

**Conclusions/Significance:**

Our results suggest that *Spiroplasma* confers protection to *D. hydei* against wasp parasitism. This is to our knowledge the first report of a potential defensive mutualism in the genus *Spiroplasma*. Whether it explains the persistence and high abundance of this strain in natural populations of *D. hydei*, as well as the widespread distribution of heritable *Spiroplasma* in *Drosophila* and other arthropods, remains to be investigated.

## Introduction

Heritable (i.e., maternally- or vertically-transmitted) associations between endosymbiotic bacteria and insects are ubiquitous [1], many of which are obligate because the host cannot reproduce without the symbiont, and thus, mutualistic because the association is beneficial to both partners. However, numerous heritable insect-endosymbiont associations are facultative (i.e., the host can generally reproduce without the symbiont), which renders the bacterial symbiont more prone to loss due to imperfect vertical transmission or due to infection costs to the host [2]. Many inherited facultative symbionts appear to reduce this risk by manipulating their host's reproduction (e.g., cytoplasmic incompatibility and son-killing) [2], but many do not manipulate reproduction [1], so their persistence must depend on other mechanisms [2]. Indeed, recent studies report heritable facultative symbionts that confer fitness benefits to their hosts, which include defense against natural enemies, tolerance to environmental stress, and increased fecundity [1], [3]. Despite the broad taxonomic diversity of inherited facultative bacteria [1], most of these mutualistic endosymbionts are clustered within the phylum Proteobacteria—e.g., the intensely-studied bacteria of aphids (class Gamma-proteobacteria; family Enterobacteriaceae) [4]–[8] and *Wolbachia* (class Alpha-proteobacteria) of *Drosophila melanogaster* [9]–[11]—, with a single example outside this group (i.e., phylum Actinobacteria; *Streptomyces* associated with digging wasps [12]).

*Spiroplasma* belongs to the class Mollicutes, an ancient wall-less bacterial group within the Gram-positive lineage [13]. *Spiroplasma* is a diverse genus that associates with many host plants and arthropods, particularly insects. Recent surveys suggest that *Spiroplasma*-arthropod associations are quite common [14]–[19], and it has been suggested to be one of the most speciose bacterial genera [20]. Within arthropods, some lineages of *Spiroplasma* transmit horizontally (usually via a plant host) while others are heritable (are transmitted vertically). Among the inherited *Spiroplasma*, a few strains that associate with flies, butterflies, and beetles are known to kill the sons of infected females [21]; a form of reproductive parasitism known as male- or son-killing. However, numerous heritable strains with undetermined phenotypes exist [15], [16], [22]–[24]. Such inherited symbionts are likely candidates for the discovery of new mutualistic associations [3].

The genus *Drosophila* is prone to associations with inherited *Spiroplasma* [16]. At least nine strains of *Spiroplasma* (defined on the basis of DNA sequence divergence and host species) have no known phenotype [24], some of which can reach relatively high frequencies of infection (above 60%) in natural populations [24], [25]. Their high prevalence and apparent lack of reproductive manipulation suggests that these endosymbionts might confer net fitness benefits to their hosts. Examination of one of these highly prevalent strains, “haplotype 1” in Mateos et al. [23], which is identical to the strain studied by Osaka et al. [19] at the three genes examined to date, suggests that: (a) it has high, but imperfect vertical transmission (95–100% at 25°C) [19]; (b) it does not affect fitness under lab conditions [19], although the fitness measures examined were not stated; and (c) it does not cause obvious male-killing [23], [25] or strong cytoplasmic incompatibility [19]. Therefore, prevalence of such an endosymbiont might be explained by alternative mechanisms such as defense against natural enemies.

Here we examine whether a highly prevalent strain of *Spiroplasma*, “haplotype 1” in Mateos et al. [23] protects its natural host *Drosophila hydei* against parasitism by *Leptopilina heterotoma*, a common parasitic wasp. *Leptopilina heterotoma* is a cosmopolitan solitary larval parasitoid of several species of *Drosophila* [26]. These wasps usually lay eggs on first- and second-instar *Drosophila* larvae. If the oviposition attack is successful, the developing wasp larva kills its host during the pupal stage, and a single adult wasp emerges from the fly puparium ∼23 days after oviposition. *Leptopilina heterotoma* overcomes the host immune response by actively suppressing host encapsulation of its eggs using Virus Like Particles (VLPs) produced within its venom glands (referred to as long glands). These VLPs enter the larval hemolymph along with the egg and rapidly bind to and enter host lamellocytes, ultimately causing them to lyse [27]. Infection by *L. heterotoma* also causes apoptosis of pro-hemocytes in the lymph gland, and possibly of the circulating plasmatocytes [28].

Because *L. heterotoma* routinely probes host larvae to determine whether they have been parasitized previously by a conspecific [29]–[31], it may be capable of detecting whether or not a fly larva is infected with *Spiroplasma*, as these bacteria reside in the hemolymph and other tissues. Similarly, since *Wolbachia* has been reported to modify *Drosophila*'s behavior [32], it is possible that *Spiroplasma* could improve the ability of its host to escape from wasp attacks. For example, enhancement of the rolling behavior response against wasp oviposition attempts [33] or stimulation of a burying response, since wasps attack at the substrate surface, could reduce oviposition rates in *Spiroplasma*-infected *D. hydei* larvae. To test this, we examined whether *Spiroplasma*-infected larvae exhibited fewer oviposition attacks than their *Spiroplasma*-free counterparts.

## Materials and Methods

### Fly strains

We collected nine wild *Drosophila hydei* females with banana baits in College Station, TX, USA (between April-Nov 2008). Each female was used to establish an independent isofemale line (hereafter fly strain)—i.e., mating only allowed among its descendants. To confirm that these fly strains were free from heritable bacteria, at least three females per fly strain were PCR-screened for infection by *Wolbachia*, *Spiroplasma* and other heritable endosymbiotic bacteria. Screening for *Wolbachia* and *Spiroplasma* was carried out on whole fly DNA extracts with primers specific to these two genera; *wsp* for *Wolbachia* (PCR conditions in Mateos et al. [23]; and *p58* [25] for *Spiroplasma* (newly –designed primers p58IV_F 5′-AAAGGTTTACATTCACCAAGTCG-3′ and p58IV_R 5′-AATTGTTCATTAACTTTATCTTGTGG-3′; annealing temperature 53°C). Screening for other heritable bacteria was carried out on ovary DNA extracts with “universal” primers for the bacterial 16S rRNA gene (primer pairs 10F–1507R and 27F–1495R) and for a 16S–23S rRNA fragment (primer pair 559F–35R); primer sequences and PCR conditions are described in Mateos et al. [23]. All PCR reactions were carried out with appropriate negative (water or DNA extraction buffer) and positive controls (*Escherichia coli* DNA extracts for the universal primers sets, and known *Spiroplasma*-positive and *Wolbachia*-positive DNA extracts for the *Spiroplasma*- and *Wolbachia*-specific primers; respectively). Throughout this study, flies were maintained on Banana-Opuntia medium at 25°C and 12 h∶12 h light:dark regime.

### Artificial infection of *Spiroplasma*

After 4–25 generations in the lab, 3–8 females per strain were artificially infected with *Spiroplasma* previously isolated from Mexican *D. hydei*. This strain is genetically identical to *Spiroplasma* strain in Kageyama et al. [25] based on previously published and new sequences of the 16S rDNA, *DnaA*, *FruA* and *FruR* genes. The donor flies were collected in 2004 and are naturally infected with *Spiroplasma* hap1 [23] and infection with other heritable bacteria was previously ruled out by PCR screening of ovary DNA extracts with primers and conditions described in Mateos et al. [23]. Artificial infection of uninfected flies was performed via adult-to-adult hemolymph microinjection using pulled microcapillaries and a manual microinjector. Infection status of the artificially infected flies was confirmed via *Spiroplasma*-specific PCR (described above) and/or examination of hemolymph under dark field microscopy.

### Antibiotic Treatment

We used antibiotics to cure *Spiroplasma*-infected flies in two cases: (1) to obtain the uninfected control for the naturally infected isoline (TEN104-102; which also served as the donor of hemolymph for all our artificial infections); and (2) for isoline 6, because our originally uninfected line was lost before completion of our experiments. We added a combination of tetracycline and erythromycin (final concentration  = 0.2 and 0.16 mg/ml, respectively) to our standard in Banana-Opuntia media for two generations. The third generation was maintained on antibiotic-free food to which we added a solution of crushed dead flies that were naturally *Spiroplasma*-free (to allow for recovery of the normal gut flora). After the third generation, the flies were maintained on antibiotic-free media with no additives. *Spiroplasma*-infection status was assessed by PCR as described above.

### Fly survival

All experiments were carried out 2–10 generations after artificial infection or antibiotic treatment of *Drosophila*. Prior to experiments, both infected and uninfected flies were maintained at low-density larval conditions (∼30 larvae/vial). For the fly survival experiment, each virgin female (≤10-days-old) was placed in a mating cage with two mature (≥10 day-old) uninfected males (from its own strain), and allowed to mate and oviposit on Petri dishes with medium. Females and males were then removed, and females were screened for infection status. Approximately 30 first instar larvae were collected from the Petri dishes and transferred to a fresh food vial. Five experienced female wasps (i.e., wasps that had been allowed to oviposit on *D. melanogaster* larvae prior to experiment), and three male wasps were placed in the vial with larvae and removed three days later. We used a single inbred highly virulent *L. heterotoma* wasp strain known as *Lh*14 [34] for all experiments. This strain was tested and confirmed positive for *Wolbachia* based on PCR assays targeting the *wsp* gene (primers and methods in Mateos et al. [23]). We recorded the number of larvae introduced to the vial, puparia, emerging flies, and emerging wasps in each vial. Puparia from which neither a fly nor a wasp emerged after 30 days were regarded as inviable. To evaluate the effect of *Spiroplasma* infection itself on fly survival rate, we also carried out the same experiment in absence of wasps. We performed 2–11 replicates per treatment per fly strain; each replicate corresponded to a separate vial. We used SAS Enterprise Guide version 4.2 statistical package to fit a Generalized Linear Mixed Model (GzLMM) with a binomial distribution of the raw data for the: (a) number of emerging adult flies/number of starting larvae (i.e., larva-to-adult fly survival rate); (b) number of emerging adult flies/total number of puparia (i.e., pupa-to-adult fly survival rate); (c) number of emerging adult flies/number of total emerging adults (flies + wasps) (i.e., adult fly emergence rate); (d) number of pupae/number of starting larvae (i.e., larva-to-pupa survival rate); and (e) number of emerging adult wasps/number of total emerging adults (flies + wasps) (i.e., adult wasp emergence rate). The independent variables were fly strain (random), *Spiroplasma* infection status (fixed), and their interaction term (random). Significance tests of random effects were based on the ratio of pseudo-likelihoods (Covtest in SAS).

### Differential oviposition

We examined whether *Spiroplasma*-infected larvae suffer significantly fewer ovipositions than their *Spiroplasma*-free counterparts. For each of three isolines (isolines 1, 34 and 57 used in experiments described above), three *Spiroplasma*-free and three *Spiroplasma*-infected female flies were individually mated with 2 uninfected males each, and allowed to oviposit for two days. Thirty first-to-second instar fly larvae were transferred into a fresh vial (three replicates  =  vials; one per female) with five parasitoid wasp females as described above. After 1.5 days, 10 out of 30 fly larvae were removed from each vial and dissected in 1 X PBS solution, to determine the number of wasp eggs per fly host. We applied a GzLMM model with a binary distribution and a logit link function to compare the presence (one or more) or absence (zero) of wasp eggs in the *Spiroplasma*-infected and *Spiroplasma*-free fly larvae. Fly strain and vial nested within fly strain were included as random factors. We also applied a GzLMM model with a Poisson distribution and a log link function comparing five categories of wasp egg numbers (0, 1, 2, 3, 4; which was the maximum number of wasp eggs observed per larva).

## Results

### Fly survival

**Figure 1:**
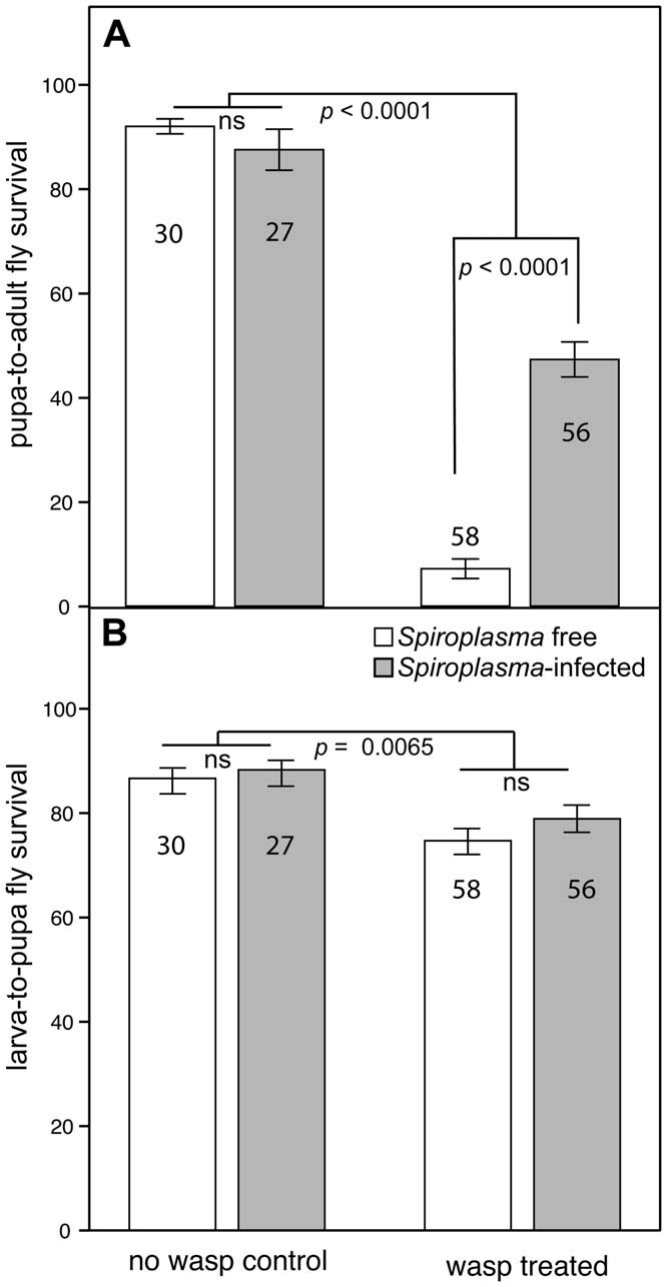
Fly survival in the presence/absence of *Spiroplasma* infection and in the presence/absence of wasp attack. Untransformed mean (±SE) survival of *Spiroplasma*-infected and *Spiroplasma*-free flies in the presence and absence of wasps. **A.** Pupa-to-adult fly survival (no. of emerging adult flies/number of pupal cases). **B.** Larva-to-pupa fly survival (no. of pupal cases/number of initial fly larvae). *P*-values are indicated for comparisons that were significantly different (ns =  not significant). Numbers within or above bars indicate number of replicates (vials) per treatment.

**Figure 2:**
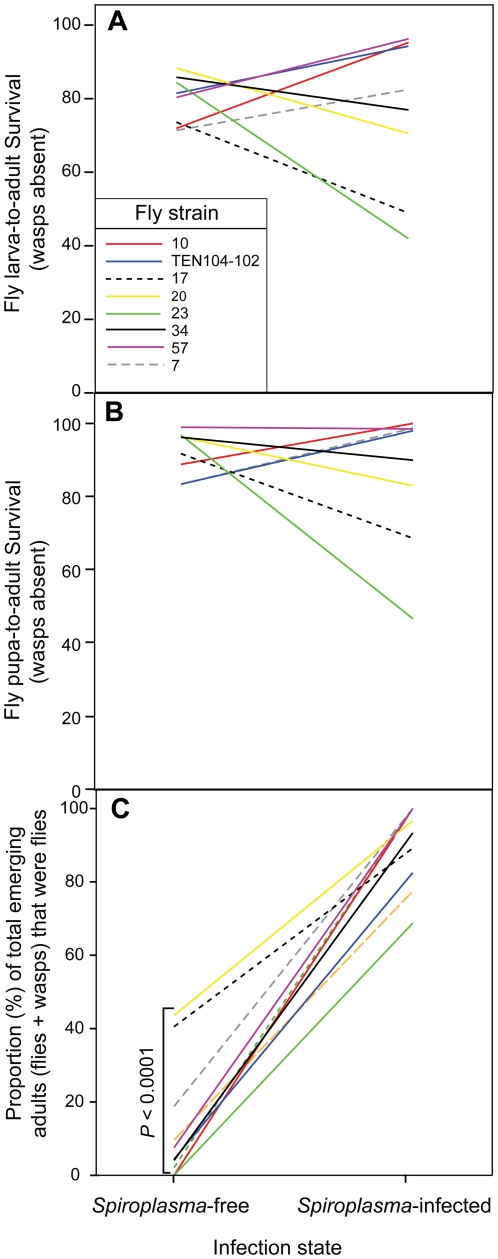
Significant interactions between infection state and fly strain in the absence (A and B) and presence (C) of wasps. Untransformed mean survival of *Spiroplasma*-infected and *Spiroplasma*-free flies. Each fly strain is represented by a different line color. **A.** Larva-to-adult fly survival (no. of emerging adult flies/number of initial fly larvae). **B.** Pupa-to-adult fly survival (no. of pupal cases/number of initial fly larvae). **C.** Fly adult emergence rate or proportion of total adults that resulted in adult flies (no. of emerging adult flies/total no. of emerging adults [flies + wasps]). (*P-*value: pseudo-likelihood ratio test “covtest” for fly strain in *Spiroplasma*-free flies only).

We compared the survival of *Spiroplasma*-infected and *Spiroplasma*-free *D. hydei* flies in the presence and absence of *L. heterotoma* parasitic wasps. We only included results from replicates in which the expected infection status of the mother was confirmed via PCR. In the absence of wasps, the effect of *Spiroplasma* infection state was not significant for any of the fly survival measures (Table 1 and Fig. 1), although the interaction between fly strain and *Spiroplasma* infection state was significant for two measures: fly larva-to-adult (*P* = 0.0082) and fly pupa-to-adult survival (*P* = 0.0002). Closer examination of these interactions (Fig. 2A) suggests that *Spiroplasma* increases larval-to-adult survival in four fly strains (7, 10, TEN, 57) and reduces it in four other strains (17, 20, 23, 34). A similar pattern was observed for fly pupa-to-adult survival (Fig. 2B). When the two groups of fly strains (those with increased and those with decreased survival) were analyzed separately, the fly strain x infection state interaction term lost significance, and the effect of infection state remained non-significant (results not shown).

**Table 1:** Effects of fly infection state (*Spiroplasma*-infected vs. *Spiroplasma*-free), fly strain (isoline) and their interaction, for each of the survival measures.

	Wasp treatment				No wasp control			
	Infection state	Mean ± SE (%)	Covariance Parameter Estimates		Infection state	Mean ± SE (%)	Covariance Parameter Estimates	
			Isoline	Isoline × infection state			Isoline	Isoline × infection state
**Fly larva-to-adult survival** (number of emerging adult flies/initial number of fly larvae)	*F*(_1, 9_) = 66.51 **(<0.0001)**	In = 37±2.98 Un = 4±1.06	0.05934 *χ^2^* = 0.61 (0.4333)	0 *χ^2^* = 0.0 (1.0)	*F*(_1, 7_) = 0.02 (0.9013)	In = 83±2.96 Un = 80±2.44	8.84E-20 *χ^2^* = 0.0 (1.0)	**0.5708** *χ^2^* = **6.98 (0.0082)**
**Fly pupa-to-adult survival** (number of emerging adult flies/total number of puparia)	*F*(_1, 9_) = 73.86 **(<0.0001)**	In = 47±3.42 Un = 6±1.62	0.1218 *χ^2^* = 1.44 (0.2301)	0 *χ^2^* = 0.0 (1.0)	*F*(_1, 7_) = 0.03 (0.8576)	In = 92±2.06 Un = 92±1.44	0 *χ^2^* = 0.0 (1.0)	**1.4141** *χ^2^* = **13.97 (0.0002)**
**Fly larva-to-pupa survival** (number of puparia/initial number of fly larvae)	*F*(_1, 9_) = 1.35 (0.2756)	In = 79±2.47 Un = 74±2.53	0.02831 *χ^2^* = 0.33 (0.5634)	0 *χ^2^* = 0.0 (1.0)	*F*(_1, 7_) = 0.13 (0.7248)	In = 89±2.46 Un = 87±2.29	0.1546 *χ^2^* = 0.83 (0.3635)	6.57E-19 *χ^2^* = 0.00 (1.0)
**Fly adult emergence rate** (number of emerging adult flies/total number of emerging adults ([flies + wasps])	*F*(_1, 9_) = 56.44 **(<0.0001)**	In = 94±2.18 Un = 11±2.60	0.2064 *χ^2^* = 0.05 (0.4092)	1.5895 *χ^2^* = 7.11 **(0.0038)**	NA		NA	NA
**Wasp “larva-to-adult survival”** (number of emerging adult wasps/initial number of fly larvae)	*F*(_1, 9_) = 69.92 **(<0.0001)**	In = 2±0.71 Un = 38±2.71	0.01492 *χ^2^* = 0.01 (0.4644)	0.07738 *χ^2^* = 0.21 (0.3248)	NA		NA	NA
**Wasp “pupa-to-adult survival”** (number of emerging adult wasps/number of puparia)	*F*(_1, 9_) = 63.72 **(<0.0001)**	In = 2±1.01 Un = 52±3.18	0.004546 *χ^2^* = 0.00 (0.4932)	0.3399 *χ^2^* = 1.82 (0.0886)	NA		NA	NA

Based on Generalized Linear Mixed Model (GzLMM) with binomial error distribution. *F*  =  F-ratio for fixed effects and corresponding degrees of freedom (subscripts in parenthesis). *χ* ^2^ for pseudo-likelihood ratio test “covtest” for random effects (*d.f.* = 1). *P*-values are shown in parenthesis (boldface: significant at α = 0.01). In  =  *Spiroplasma*-infected; Un  =  *Spiroplasma*-free.

In the presence of wasps, fly larva-to-adult survival, fly pupa-to-adult survival (Fig. 1A), and fly adult emergence rate (i.e., number of adult flies/number of total adults including wasps) were significantly higher in the *Spiroplasma*-infected flies than in the *Spiroplasma*-free flies (Table 1). Similarly, as expected, wasp survival was significantly lower in *Spiroplasma*-infected treatments (Table 1). A significant interaction between fly strain and *Spiroplasma*-infection state was observed for fly adult emergence rate (*P* = 0.0038; Table 1), but all fly strains exhibited an increased survival in the *Spiroplasma*-infected treatment (Fig. 2C). The protective effect of *Spiroplasma* is striking, but not complete. In the absence of wasps, fly pupa-to-adult survival averaged ∼89.87% for both, *Spiroplasma*-free and *Spiroplasma*-infected flies (Fig. 1A). In the presence of wasps, the survival of *Spiroplasma*-free flies averaged ∼7.17%, but survival of *Spiroplasma*-infected flies was much higher (∼47.30%). In contrast, larva-to-pupa survival was not significantly affected by *Spiroplasma* infection in the presence of wasps, (Table 1; Fig. 1B). Nevertheless, the presence of wasps is correlated with higher fly mortality (lower larva-to-pupa survivorship) during the larva-to-pupa stage [wasp effect *P* = 0.0065; GzLMM including wasp treatment, infection state, and fly strain (random effect); Fig. 1B]. Therefore, our results suggest that *Spiroplasma* protects the fly against wasp-induced mortality, but that the effects of this protection are only reflected in differential fly survival during the pupa-to-adult transition.

### Differential Oviposition

**Figure 3:**
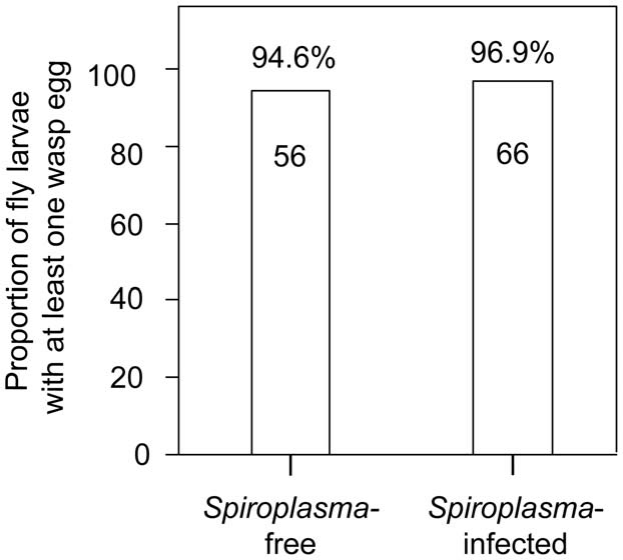
Wasp oviposition frequency in *Spiroplasma*-free and *Spiroplasma*-infected *Drosophila hydei* larvae. Proportion of fly larvae that had at least one wasp egg. Numbers above columns  =  exact proportion. Numbers in columns  =  number of fly larvae examined.

Our fly and wasp survival measures suggest that *Spiroplasma* infection confers protection to *D. hydei* against wasp-induced mortality, and that this effect is not detectable before the fly pupal stage. Nevertheless, we tested whether this protective effect could be also mediated by a pre-oviposition mechanism, in which *Spiroplasma*-infected larvae suffer significantly fewer ovipositions than their *Spiroplasma*-free counterparts. The number of wasp eggs found per fly larva did not differ significantly between *Spiroplasma*-free and *Spiroplasma*-infected treatments, for either the GzLMM with Poisson distribution (*F*_(1,112)_  = 0.89; *P* = 0.3476) or the GzLMM with a binary distribution (i.e., one or more wasp eggs grouped into a single category: *F*(_1,112_)  = 0.40, P = 0.5248; see Fig. 3). No significant effects of fly strain or vial were detected. These results suggest that *Spiroplasma* infection does not protect fly larvae from wasp oviposition attacks.

## Discussion

Our results indicate that *Spiroplasma* confers protection to its fly host against wasp-induced mortality during the pupal-to-adult transition. Although there appears to be significant variation among fly strains in the degree of protection induced by *Spiroplasma*, indicated by the significant *Spiroplasma* infection state x fly strain interaction for fly adult emergence rate (Table 1), evidence for *Spiroplasma* protection was found in every fly strain examined. This interaction could be due to genetic variation in “natural” (i.e. not *Spiroplasma*-mediated) resistance, as suggested by the significant effect of fly strain on survival against wasp attack in the absence of *Spiroplasma* infection (χ^2^ = 53.16; *P<*0.0001; *d.f.* = 1; see Fig. 2C). Indeed, variation in *Drosophila* natural resistance to parasitoids has been reported [35]. The infection x fly strain interaction could also result from variation in the original transfection itself (e.g., different transfection hemolymph volumes or bacterial titers, or physical damage to transfected female) rather than host genetic background. Despite the effect of host background and possibly transfection, *Spiroplasma* infection increased survival in the presence of wasps for all fly strains. Although infection by *Spiroplasma* does not restore survival to the levels observed in the absence of wasps, the degree of protection is high and potentially important in nature (although this remains to be tested). Furthermore, it is possible that protection is actually greater than shown here, as we cannot be certain that all of the larvae included in the *Spiroplasma*-infected treatments were infected, because bacterial transmission is less than 100% efficient [19]. We assessed *Spiroplasma*-infection status via PCR for a subset of the emerging adult flies (∼200 individuals) from the wasp-treated *Spiroplasma*-infected experiments. Indeed, 100% of these flies were *Spiroplasma*-positive, further supporting the notion that *Spiroplasma* confers protection against *L. heterotoma*, since no *Spiroplasma*-free survivors were observed in these vials.

At this stage the protective mechanism is unknown, but it is exerted after wasp oviposition and is detectable as differential survival during the pupa-to-adult transition. Several post-oviposition mechanisms of protection by *Spiroplasma* are possible, for example: (a) reduced availability of resources necessary for wasp development (if *Spiroplasma* consumes resources that would otherwise be available for the parasitoid); (b) the presence of a substance toxic to the parasitoid; and/or (c) an enhanced immune response of the fly larva against the parasitoid (e.g., by countering the encapsulation-suppressive effect of the parasitoid venom or by increasing production of lamellocytes). Presence of a toxic substance encoded by a bacteriophage is the hypothesized protective mechanism provided by the Gamma-proteobacterium *Hamiltonella defensa* to its aphid host against a parasitoid [36], [37]. In this regard, *Spiroplasma* strains associated with several species of *Drosophila*, including a non-male killing strain of *D. hydei*, are known to possess bacteriophages with lytic activity [22], [38], [39]. Whether these viruses play a role in the defense against wasp larvae in this system remains to be explored. In this study, we observed no evidence of melanotic capsules in surviving flies, which suggests that the protective mechanism is not through an enhanced fly immune response to the parasitoid. However, although melanotic response is an indication of encapsulation, examples exist of parasitoid larvae that have been killed in the absence of a melanotic response [40], so further work would be needed to study this possible mechanism.

Whether or not the protective effect of *Spiroplasma* reported here explains the long-term persistence of this *Drosophila-Spiroplasma* association remains to be tested and depends on, among other things, the degree of *D. hydei* mortality caused by *L. heterotoma* under natural conditions, the reproductive fitness of *Spiroplasma*-infected flies that survive a wasp attack, the degree of protection conferred by *Spiroplasma* across different host, symbiont and parasitoid backgrounds (e.g., host x parasitoid and symbiont x parasitoid interactions) [41], and any fitness costs associated with *Spiroplasma* infection. Future studies to resolve this issue are warranted. Nevertheless, the high prevalence (>60%) of *Spiroplasma* infection in two geographically distinct populations of *D. hydei* found in Arizona [24] and Japan [25]. the cosmopolitan distribution of *L. heterotoma*, and the high level of *Spiroplasma*-free *D. hydei* mortality when attacked by *L. heterotoma* in our experiments, strongly suggest that the protection conferred on *D. hydei* by *Spiroplasma* in nature may be significant.

Our study has important implications. First, given the rising number of reports of *Spiroplasma* in arthropods [13]–[17], [23], [42]–[49], particularly in the genus *Drosophila*, most of which have undetermined effects [15], [16], [22]–[24], there is a high probability of discovering defensive mutualisms in different combinations of host-*Spiroplasma* lineages. Second, our discovery opens the possibility of using the *Drosophila* model system to study defensive mutualisms. Third, our findings could have important implications in studies that use the *Drosophila*-parasitoid model system, such as research on cellular immunity [40], behavior [50], and evolutionary ecology [35]. Fourth, if protection against parasitoids is a general feature of maternally-transmitted *Spiroplasma* in insects, it could have important implications for biological control, because parasitic wasps are the most successful group of biological control agents [51], and an increasing number of insects are reported to be infected with *Spiroplasma* symbionts. Fifth, our study significantly expands the taxonomic range of both the inherited defensive symbionts (to include the wall-less class Mollicutes of the phylum Firmicutes) and also the hosts protected against parasitoids by an endosymbiont (to include Diptera).
